# Fostering experiential learning of neurocognitive skills in brain injury tele-rehabilitation: bridging gaps in remote training by integrating scenario-based digital experiences with coaching

**DOI:** 10.3389/fnhum.2025.1593246

**Published:** 2025-05-12

**Authors:** Fred Loya, Deborah Binder, Nicholas Rodriguez, Bruce Buchanan, Tatjana Novakovic-Agopian, Anthony J.-W. Chen

**Affiliations:** ^1^Department of Veterans Affairs, Veterans Affairs Northern California Health Care System, Veterans Health Administration, Mather, CA, United States; ^2^Northern California Institute for Research and Education (NCIRE), San Francisco, CA, United States; ^3^Department of Veterans Affairs, VA Palo Alto Health Care System, Veterans Health Administration, San Francisco, CA, United States; ^4^Department of Psychiatry, University of California, San Francisco, San Francisco, CA, United States

**Keywords:** traumatic brain injury (TBI), brain state, cognitive rehabilitation, tele-rehabilitation system, experiential learning, executive (dys)functions

## Abstract

Dysregulation of brain state is common following traumatic brain injury (TBI), contributing to long-term difficulties in important life pursuits, spanning school, work, and beyond. Brain state dysregulation makes it difficult to effectively organize and direct cognition and behavior to accomplish any number of goals, resulting instead in difficult-to-understand combinations of neurocognitive and emotional symptoms: distractibility, forgetfulness, poor follow-through, irritability, reduced frustration tolerance, and becoming easily overwhelmed. Given underlying heterogeneity with neurocognitive-emotional symptoms, it may be highly efficient to train use of state regulation skills (SRS) as a generalizable approach to facilitate processing of neurocognitive demands encountered along individual goal pathways. In this report, we present an innovative system of *guided experiential skill* learning of goal-directed SRS – one that rationally integrates experiential digital technology designed to practice applying and integrating SRS directly into goal-based functional challenges with therapist-led training to maximize skill learning, transfer, and generalization. Moreover, we designed this system specifically to bridge important gaps that interfere with skill learning when individuals are remote from therapists. To advance the integration of technology into rehabilitation neuroscience, we present this communication as a hybrid of intervention design (introducing principles and features), “user experiences” (sharing vignettes informative of the value of technology integration into the learning process), and a controlled, proof-of-principle pilot intervention study for a small cohort of individuals (*n* = 18) with chronic TBI (assessing the plausibility of strengthening goal-directed functioning, as indexed by performance on neurocognitive assessment tasks and complex functional tasks, as well as ratings of personal life functioning). Data suggest that a *technology-augmented remote guided experiential learning approach* may bridge important gaps in skill learning to help individuals improve goal-directed functioning. This line of work will inform further advances in remote neuro-cognitive rehabilitation.

## Introduction

For many individuals, the long-term impact of traumatic brain injury (TBI) on day-to-day neuro-cognitive functioning can be significant as well as difficult to address therapeutically. This is particularly the case when such injuries co-exist with experiential trauma and/or other conditions that add to dysregulated emotions, as is often the case for military Veterans and others experiencing post-traumatic stress (Chen and Loya, 2019; Chen et al., 2020; Vasterling et al., 2009; Sayer et al., 2009). While functional cognitive difficulties may not always be easily detected with conventional assessment methods (Howieson, 2019; Neale et al., 2022; De Souza et al., 2024), many of these individuals nevertheless struggle in critical life domains, such as at school (Elnitsky et al., 2018) and work (Benedictus et al., 2010) and with multiple aspects of self-management (Donald et al., 2022; Brenner et al., 2023; McGarity et al., 2017). In fact, functional difficulties may actually worsen over time (Wilson et al., 2017; Mac Donald et al., 2017; Mac Donald et al., 2021), presenting clinicians with the difficult task of helping to unravel tangles of sequalae that have accumulated over many years (Chen and Loya, 2019; Sayer et al., 2009). An alarm has been raised (Dams-O’Connor and Tsao, 2017); advances in therapeutics are sorely needed.

One potential high value target for TBI rehabilitation is strengthening goal-directed regulation of brain state (Chen and Loya, 2019; Chen et al., 2020; Chen and Loya, 2014; DeBettencourt et al., 2015; McCormick et al., 2020). Conceptually, brain state reflects neuronal activity patterns associated with physiologic processes that, in turn, serve as the foundation underlying all cognitive activity (McCormick et al., 2020; Greene et al., 2023). Brain state and its associated functional circuitry requires dynamic adjustments and coordination to facilitate goal-directed functioning as aligned with the action context at any given time (Flavell et al., 2022; Harris and Thiele, 2011). For example, researchers have identified dynamic fluctuations to brain state associated with cognitive performance (Taghia et al., 2018; Cai et al., 2024) and that distinguish healthy patterns of cognitive functioning from attention dysregulation (Cai et al., 2024; Cai et al., 2021b; Mizuno et al., 2022), as well as associations between developmental trajectories of brain state and later executive functions (Ye et al., 2024). Furthermore, parameters of brain state influence response to interventions aimed at improving cognitive-emotional functioning (Sack et al., 2024), including behavioral training involving learning new skills (Arnemann et al., 2015).

On the other hand, dysregulated brain state is increasingly understood to contribute to various forms of psychopathology (Greene et al., 2023; Phalip et al., 2024; Rowland et al., 2024; Scharfen and Memmert, 2024), including TBI-associated dysfunction. Following injury to the brain, one’s ability to effectively regulate brain state can become compromised (Chen et al., 2020; Chen and Loya, 2014; Novakovic-Agopian et al., 2021; Novakovic-Agopian et al., 2018), resulting in difficulties navigating and responding to ever-changing life circumstances and conditions, including not just those associated with the environment or a specific goal or task, but also originating from within the individual (e.g., fluctuations in attention). That is, difficulties with regulating brain state may contribute to problems directing attention, processing and remembering key information, managing disruptions, and effectively problem-solving – common cognitive symptoms following TBI that often co-occur with dysregulated emotions, such as feeling overwhelmed, stressed, flustered, anxious, and irritable (Chen et al., 2020; Chen and Loya, 2014; Vasterling et al., 2018; McInnes et al., 2017; Konrad et al., 2011). Of note, with poorly regulated brain state, individuals may not only be less efficient processing individual neuro-cognitive demands, but may be especially inefficient, variable, or ineffective with integrating and coordinating multiple neuro-cognitive functions required for achieving complex goals. This may help account for why cognitive-emotional challenges to goal-direction become magnified when goal activity must be coordinated and maintained over extended periods of time.

Regions of prefrontal cortex that are directly involved with goal-directed functioning (Corbetta et al., 2008; Japee et al., 2015) and implicated in TBI-associated cognitive dysfunction (Xiong et al., 2016; Lu et al., 2020) appear critical to the effective regulation of brain state (Cai et al., 2024). Furthermore, prefrontal cortex does not operate in isolation – as an important principle, prefrontal regions engage dynamically and variably with posterior regions in functional networks to achieve goal-directed control over various specific functions as pertinent to each specific context (Menon and D’Esposito, 2021; Cai et al., 2021a). Thus, engagement of different networks will depend on not only the domain-general cognitive process demands (e.g., working memory), but also the specific information content, modality (e.g., auditory or visual), action plan and context (Chen et al., 2006; van Ede et al., 2019). In short, brain state forms a critical foundation supporting multiple aspects of cognition and behavioral functioning.

These findings and other considerations have direct implications for the design of cognitive rehabilitation interventions to achieve effective skill learning, transfer and generalization. In particular, it is important to consider how improving regulation of brain state (or any neurocognitive skill) may help individuals pursue functional goals in everyday life contexts. Such contexts frequently require active and dynamic cognitive work along extended goal pathways – that is, longitudinal and complex chains of neurocognitive processes embedded in sequences of actions that need to be directed to achieve goals, while in the setting of potentially disruptive events. Traversing these pathways presents numerous opportunities for a dysregulated brain state to result in failure and derailment. Systematic practice with goal-directed regulation of brain state to *explicitly* facilitate neurocognitive processing is especially important if we are to achieve useful skill application in real life contexts where integration of skills is difficult.

One particularly salient vulnerability to regulation of brain state along goal pathways is posed by unanticipated disruptions; many with TBI have difficulty resuming or re-engaging goal activity when taken off track. Whether working in a restaurant, going to school, or completing an errand at home, it is not uncommon for disruptions to cause someone with TBI to go completely off track (Chen and Loya, 2019; Whyte et al., 2000) – even by something as relatively innocuous as a text message. *Disruptions* not only capture neural resources needed for pursuing goals, such as those involved with attention and processing information, but also frequently evoke strong emotional responses, like anger and frustration, that may also derail goal activity. This may be the case because successfully managing disruptions requires that multiple neural-cognitive resources are effectively and dynamically coordinated across both the primary goal and the disrupting event: one’s goal and associated action plan (goal pathway) must, first, be clearly represented in memory; next, neural-cognitive resources must be *re-directed* to the disruption; and finally, these resources need to be *re-directed* back to the original goal pathway so that goal activity can be resumed. This last process requires sub-processes of retrieving and re-activating the original goal representation and then re-starting activity at the appropriate next step on the goal pathway. Thus, training that emphasizes state regulation (SR) to manage disruptions along goal pathways may be particularly helpful with promoting goal-directed functioning following TBI.

We argue that innovations in training tools and intervention methods could facilitate *learning* how to actively and intentionally utilize state regulation skills (SRS) to improve goal-directed functioning in daily life. On the one hand, this would require, as a basic foundation, helping individuals practice applying SRS in the context of *goal-based challenges* of varying kind, intensity, and complexity. Experiential learning theory (Kolb et al., 2001) takes as axiomatic that concrete experiences not only provide contextually rich and embodied learning environments necessary for the acquisition of new skills, but also help to deepen learning by facilitating experimentation, self-discovery, and abstraction of generalizable lessons relevant to new contexts and settings. That is, training across modalities and situations provides ample opportunities for *learning by doing,* including facilitating training domain-general core control processes (D’Esposito and Chen, 2006). On the other hand, individuals would also benefit from various supports to maximize skill learning, especially regarding integrating skills into daily life goals and situations. Active and ongoing support, guidance, and feedback are generally facilitative of learning (Alfieri et al., 2011), and these factors may be especially relevant for TBI rehabilitation (Duncan et al., 1995; Toglia and Kirk, 2000). Within the context of learning how to effectively regulate state to improve goal-directed functioning in everyday life, for example, individuals with TBI may benefit from help with understanding the relevance of the SR concept to their specific goals and challenges; identify opportunities along individual goal pathways to apply SRS; establish plans for using SRS in these instances; and problem-solve any issues arising with skill use. Taken together, we contend that for individuals with TBI to learn how to effectively regulate state to improve goal-directed functioning, they would benefit from an approach that supports *guided experiential learning* via training that integrates opportunities for robust and varied skill practice with active, ongoing support.

The thoughtful design and integration of digital tools into therapist-led training may help achieve this training objective. A technology-augmented approach to SRS training also has the potential of being *remotely deployable*, helping to increase access to cognitive rehabilitation services for individuals with TBI. Indeed, several features of digital technologies are particularly well-suited to support remote experiential learning of SRS. First, goal-based challenges can draw from real-life to be specifically designed to engage various neurocognitive processes in a range of distinct goal pathways. For example, digital scenarios can be designed as extended goal pathways that integrate (rather than isolate) selective attention, working memory, and other cognitive control processes, and also incorporate *disruptions* to interfere with cognitive processing along these pathways. This resultant setup would allow for robust opportunities to practice SRS at key cognitive junctures in context, as well as facilitate intensive, systematic, and frequent (on demand) skill practice. Achieving variability and intensity of practice is not only helpful with developing skills, but may also be critical for skill generalization and transfer (Toglia et al., 2010; Raviv et al., 2022; Moshon-Cohen et al., 2024).Variability of training exercises may be particularly helpful when the intent is to improve multi-modal abilities such SR in relation to attention, working memory, and other executive functions subserved by networks involving prefrontal cortex (D’Esposito and Chen, 2006). Of note, curating these types of calibrated training opportunities is difficult, if not impossible, in naturalistic settings, especially via tele-rehabilitation, without technology.

Second, digital scenarios allow for challenges to be introduced in a progressive manner, with complexity increasing over the course of training. Here, complexity can reflect several distinct qualities, such as workload demands, demands for coordination of multiple neurocognitive processes, and variable demands imposed by disruptions. In other words, digital experiences can be designed to help individuals learn to apply skills across a range of complexities and demands. Furthermore, all these various challenge parameters can be strategically tuned to optimize skill learning *on an individual basis*, allowing for the adaptation of specific cognitive processing demands separately from the broader challenge context in which those demands are embedded. This degree of control and calibration is also difficult to achieve with traditional rehabilitation tools and methods.

Third, with the intent of individualizing training experiences, digital scenarios can provide individualized data-driven feedback to facilitate skill learning and inform coaching efforts. Various aspects of skill use (i.e., quantity of use, where a skill is applied along a goal pathway) can be monitored and analyzed in relation to performance metrics, helping to establish relationships between skill use and therapeutic benefits. One major advantage of this setup is that it addresses a typical gap that occurs when evaluating benefits of any skills training – reliance on retrospective self-report to determine efficacy of skill use. While such reports are not uninformative, they are subject to biases and distortions (Iverson et al., 2010).

Fourth, digital technologies allow for incorporation of other supports that can facilitate training overall. This includes the strategic cueing of skill use during early phases of training, followed by fading of cues over time as well as contingent activation of cues based upon individual needs (e.g., if multiple opportunities are missed). In addition, tutorials and digital guides can model skill use in relation to specific challenges, helping to reinforce concepts introduced during training sessions. Moreover, digital technologies allow for design and delivery of strategic rewards in engaging environments to reinforce skill learning goals (Nieto-Escamez et al., 2024; Chen et al., 2017).

Finally, and of great clinical relevance, digital technologies can be designed to help advance *remote* TBI rehabilitation. While remote TBI rehabilitation is increasingly utilized in health care settings, remote interactions themselves remain limited for skill training purposes. For instance, Ng et al. (2013), in one of the few tele-rehabilitation studies of executive function training, reported that the remote format interfered with their ability to observe, analyze, and provide corrective feedback on skill use, factors that they felt reduced the effectiveness of their training and contributed to an over-reliance on trainees’ retrospective reports to inform treatment planning. The integration of experiential technology into remote rehabilitation may help address this gap in the skill learning and generalization process. Ultimately, design and integration of technology to support skill learning may help extend the reach of remote TBI rehabilitation and strengthen its impact.

Previously established approaches to remote cognitive rehabilitation have not addressed the above identified needs for training *goal-directed SR*, leaving critical gaps in need of bridging. On one end of the spectrum of interventions, conventional SR approaches are largely based upon mindfulness and meditation practices (Jha et al., 2007) that train SR in relative isolation from the neurocognitive challenges and goal pathway contexts in which individuals are prone to dysregulation. That is, these practices are not experiential, goal-based approaches for training applied SRS. Individuals with TBI would benefit from training SR applied and integrated into goal actions, in contexts in which they are vulnerable to dysregulation and derailment. This is hypothesized to be especially valuable in the context of unanticipated disruptions and other events that require adjustments to cognitive processing “in-the-moment.” On another end of the spectrum of intervention approaches, training based on task practice has been extensively studied, and there are now many available computerized task-based trainings (Simons et al., 2016). These approaches generally have individuals repetitively engage in digital challenges designed to isolate select neurocognitive functions from a larger goal context. Thus, these trainings do not attempt to cultivate SR at all, but instead intend to improve the functioning underlying repetition and practice. This approach has been well documented to result in improvements in the practiced tasks and similar assessment tasks, but studies have thus far shown limitations in generalizability of effects to non-trained tasks and real-world functioning (Simons et al., 2016; Shipstead et al., 2012; Melby-Lervåg and Hulme, 2013).

Training dynamic SR to improve goal processing may benefit from *experiential training* augmented by technology. Learning to apply SRS flexibly in real-world goal contexts requires individuals to not only be able to understand that SRS have a relationship with cognitive functioning, but also strengthen their actual abilities to apply SRS when and where needed most to overcome challenges and achieve their intended outcome. While other research interventions, including our previous work (Novakovic-Agopian et al., 2021; Novakovic-Agopian et al., 2018), have provided some conceptual introductions to SR in relation to cognitive functioning, we are not aware of prior interventions that have specifically designed training to experientially and extensively train SRS as applied and integrated into cognitive processing during work toward goals, across a range of experiences.

The purpose of this report is to present an innovative approach for *guided experiential learning* of goal-directed SRS designed for use in remote rehabilitation. We designed and developed digital scenarios, inspired by real-life challenges, to function as concrete opportunities to practice SRS. Challenges required the integration and coordination of multiple neurocognitive processes along extended goal pathways, including disruptions to goal-direction. In addition, we designed digital scenarios to track key aspects of skill use, quantifying skill application as well as its impact on indices of functional performance, facilitating a process of data-driven feedback to support skill learning. We integrated this experiential technology into a system of remote coaching via tele-video to help guide skill development and extend skill use to goals and situations in personal life. Overall, we designed this technology-augmented approach to support the application and integration of SRS into a range of experiences, spanning digital scenarios to personal life goals and settings. We further designed this approach to be remotely deployable and to maximize skill learning, transfer, and generalization.

Intervention development involved a combination of digital technology, experiential design, cognitive rehabilitation training, and coaching linked to implementation testing and clinical pilot testing. We, therefore, present a hybrid report intended to support the growing field of technology integration into rehabilitation interventions. We present key principles and features influencing the design of this technology-augmented, guided experiential skill training. We share observations and discoveries from “user experiences” (from trainees and trainers), with a particular focus on how designed experiences support skill learning objectives, illustrated via clinical vignettes. Finally, we report on a controlled pilot intervention study that examined intervention effects on goal-directed functioning, and we contextualized these observed changes in relation to treatment-as-usual. We evaluated training-associated changes across multiple levels of functioning, important for understanding potential relationships (or disconnects) between individual neurocognitive processes, integrated functional performance, and experience of functioning in naturalistic settings. Together, these experiences and data are intended to inform the advancement of remote, technology-augmented neurocognitive skill training to improve personal life functioning.

## Methods

### Participants

Eighteen participants (*n* = 15 military veterans; 83% male; 78% Caucasian; average age = 41.3 years; average education = 14.9 years) completed all assessment and training activities. Study inclusion criteria included the following: (1) having history of mild and/or moderate TBI per American College of Rehabilitation Medicine criteria. History of TBI was confirmed via structured clinical interview conducted by a clinician experienced in the diagnosis of TBI, and when available, review of medical records; (2) being in the chronic phase of injury (i.e., > 6 months post-injury); (3) being medically stable, including a stable medication regimen; and (4) having current cognitive complaints that reportedly interfered with functioning in at least one major life domain. All participants endorsed at least one of the cognitive symptoms on the neurobehavioral symptom inventory (Cicerone and Kalmar, 1995) being present to a “moderate” degree or greater. No participant had major psychiatric, substance use, or other neurologic conditions that limited their participation; however, presence of behavioral health comorbidities, such as depression and PTSD, were permitted. Participants were allowed to continue to participate in routine clinical care throughout their involvement in the study. All participants were independent in basic activities of daily living.

Participants were recruited via VA clinical referrals and advertisements placed at VA and University of California. Following obtaining consent per IRB-approved procedures, participants were randomly assigned to intervention conditions (remote training or treatment-as-usual). However, one individual assigned to the remote training condition could not participate due to other time commitments and was allowed to participate in the treatment-as-usual condition. A summary of participant characteristics is provided in Table 1. The groups appeared generally well-matched except that participants in treatment-as-usual were slightly older.

**Table 1:** Summary of participant demographic and injury-level characteristics by intervention and treatment-as-usual conditions.

Variable	SRS training (*n* = 8)	Treatment-as-usual (*n* = 10)
Demographics		
Age, years: *M* (*SD*)	35.13 (11.74)	46.40 (8.81)
Education, years: *M* (*SD*)	14.38 (1.85)	15.30 (2.87)
Gender: Male (*n; %*)	6 (75%)	9 (90%)
Ethnicity: Caucasian (*n; %*)	7 (88%)	7 (70%)
Injury characteristics		
Number of Injuries: *M* (*SD*)	2.25 (1.17)	1.6 (0.97)
Time since worse injury, years: *M* (*SD*)	9.88 (9.61)	13.10 (10.30)
Self-report of functioning		
NSI, cognitive subscale: *M* (*SD*)	9.50 (3.12)	11.11 (3.30)

NSI, Neurobehavioral Symptom Inventory; SRS, State Regulation Skills.

### Intervention principles and framework

#### Key principles

Training centered on developing a core skill to facilitate *goal-directed SR* – that is, the intentional and habitual practice of regulating state to optimize cognitive-emotional functioning while actively working on one’s goals. To help with both skill development and generalization to personal life, training was designed around a framework emphasizing *state regulation skills applied and integrated into a range of experiences* (SR-AIRE), starting with digital challenge scenarios and building toward everyday goals and life situations. Key principles of SR-AIRE included intensive practice with applying skills directly to specific cognitive processes involved with work toward goals as well as integrating skills into the larger goal context (pathway) in which this work is performed; achieving a sufficient degree of repetition across different goal contexts to train skill application as an automatic response to goal-based challenges; developing strategic intentions for implementing skills along goal pathways; and providing multi-level feedback and active guidance to optimize learning and promote skill use in personal life. To help actualize these principles in practice, features to bridge potential gaps in skill learning and generalization across individual participants were also emphasized. This included helping to deepen conceptual understanding and insight regarding how cognitive-emotional processes can affect work toward goals; identifying opportunities for skill use across myriad goal contexts and pathways (ranging from the hypothetical to the personal); and encouraging exploration and experimentation with skill use to maximize learning on an individual basis.

#### General organization

**Figure 1:**
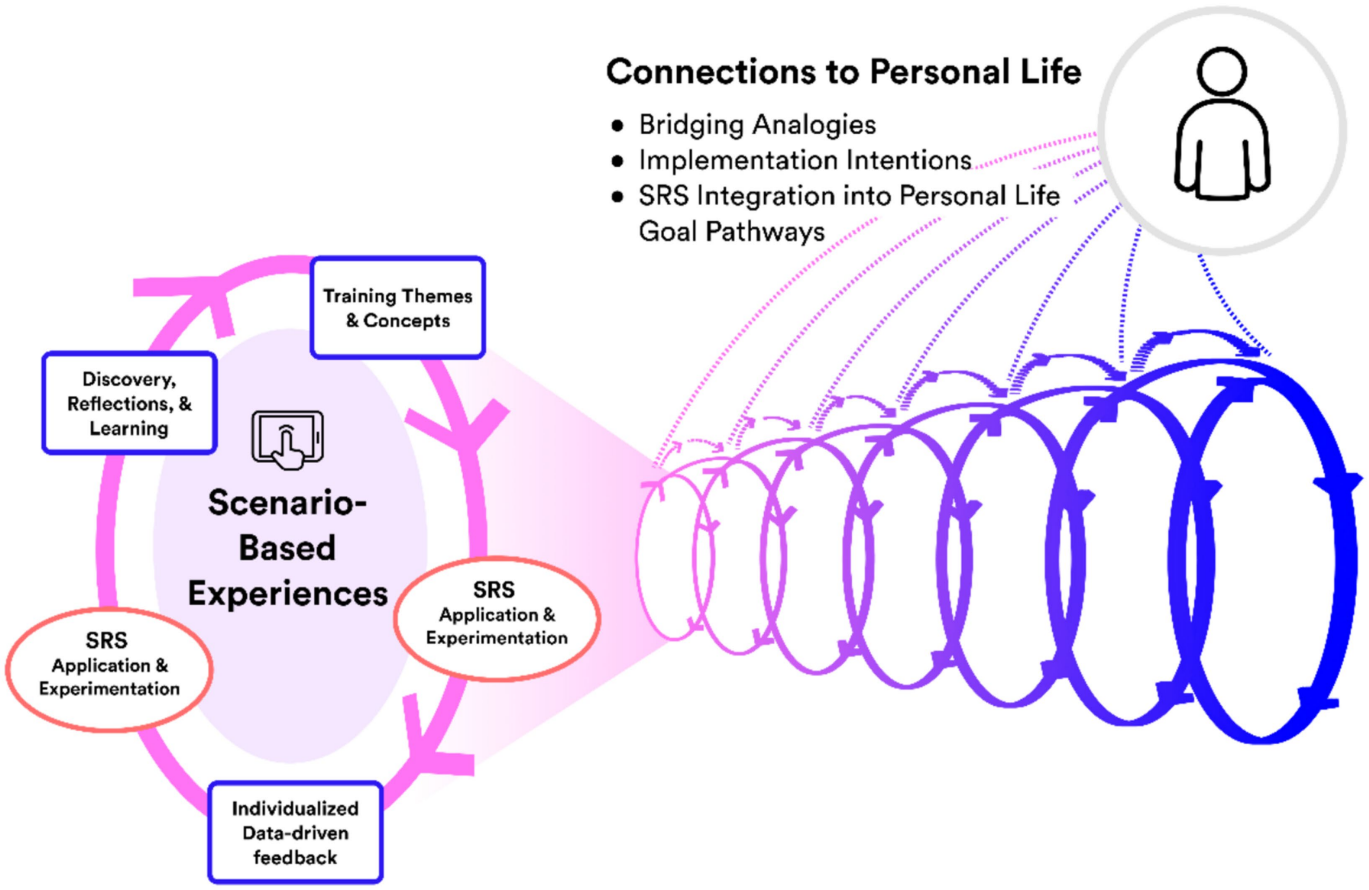
Conceptual model of technology integration into remote SRS training. Scenario-based experiences support SRS application and integration into a range of experiences (SRS-AIRE), while remote trainers help to abstract learnings from these experiences applicable to personal life goal pathways. This framework involves deepening loops of learning intended to bridge training experiences to daily life.

The above features were integrated into a training system designed to function as interlocking *loops of learning.* As depicted in Figure 1, each *learning loop* followed a general, multi-stage learning process, centered on a unique scenario-based digital challenge. Interactive scenarios anchored each training (loop) session by providing participants the means to explore training concepts and themes experientially as well as directly practice using SRS in relation to embedded neurocognitive challenges, all while receiving multiple levels of guidance and feedback. Learnings derived from each loop of experience were intended to be “carried forward” to subsequent training experiences, including to personal life situations. The intention behind this approach was for participants’ understanding of the SR concept and their ability to apply and integrate SRS into goal pathways would progressively deepen over training, leading ultimately to improved SR while pursuing everyday goals in life contexts.

Each learning loop emphasized the following:

- Facilitating conceptual understanding of training content. Training concepts (e.g., state regulation, goal pathways) first introduced in didactic format were elaborated upon and illustrated via interactions with digital scenarios. Trainers first worked with trainees to conceptualize cognitive activity in digital scenarios from within a goal pathway framework before extending this conceptualization to their own personal goals. For example, digital challenges requiring trainees to hold information in mind while managing disruptions resulting in frustration was first conceptualized via the goal pathway concept and the theme of brain SR; this concept then was connected with individual experiences that mirror this process, such as trying to complete tasks at work while addressing disruptions from co-workers. One goal of this process was to help participants identify potential points of intervention (e.g., SRS use to overcome situational demands, in response to internal experiences).
- Developing skills through experiential practice. Participants practiced applying SRS in a variety of challenge contexts, starting with digital scenarios and building toward personal life. Digital scenarios provided efficient means for curating opportunities to systematically and intensively practice skills in relation to embedded neurocognitive demands of increasing complexity.
- Fostering learning through experimentation, discovery, guidance, and feedback. A critical goal of training design was to help establish direct links between SRS use and positive changes to goal-directed functioning; highly relevant to this process was identifying specific junctures along goal pathways where individuals are prone to dysregulation and, therefore, could benefit from skill use. To help achieve this degree of insight and self-awareness, participants were encouraged to adopt an *experimental framework* to explore how to most effectively utilize SRS at different cognitive junctures along goal pathways on an individualized basis. For example, this framework enabled trainees to evaluate the effects of applying SRS to facilitate encoding of information by comparing performance on trials in which they applied the skill to encoding challenges vs. trials in which they did not. These training experiences were reviewed in detail each week, in which trainees’ subjective experiences and personal observations were combined with review of empirical data on SRS use in digital scenarios. Particular emphases were placed in these reviews on examining instances in which objective performance data and trainees’ subjective experiences were misaligned, as well as problem-solving skill use. For example, trainers helped identify opportunities in the form of difficult challenges in which they did not apply SRS, such as when having to switch between different scenarios, and helped them to establish plans for using SRS at that juncture in future instances, both in relation to digital scenarios and personal life. Thus, a major goal of this review process was for participants to gain insight into the various ways that improved SR may benefit their goal-directed functioning, as well as identify ways to optimize SRS use.
- Building bridges from digital scenarios to personal life. As a final step, training emphasized applying SRS to everyday goals and life situations. To help extract generalizable insights from training experiences relevant to each trainee’s individual goals, trainers utilized digital scenarios as *experiential analogies;* this process involved identifying how learnings from digital scenarios might generalize to various contexts and challenges, ranging from hypothetical scenarios to each individual’s specific goals and difficulties. Trainees received support with identifying specific junctures along goal pathways in daily life where skills could be utilized, establishing plans for applying skills at those instances (i.e., making use of *implementation intentions;* see below), and reviewing implementation experiences to gain further insight into the skill use process.

In sum, training incorporated principles derived from models of experiential learning (Kolb et al., 2001) and guided discovery-based learning (Alfieri et al., 2011): participants experimented on an individual basis with applying SRS in digital scenarios; reflected upon these experiences with the aid of data and trainer input; abstracted generalizable lessons relevant to personal life goals and situations; and practiced implementing SRS in these individual settings. Insights, skills, and learnings from each training experience informed subsequent challenges and experiences.

#### Specific training components

The following specific components were designed and incorporated into the structure of the intervention. (1) *Scenario-based experiences* were designed to form distinct (a) *goal pathways* consisting of various embedded neurocognitive challenges, including adaptive variations to (b) *information load* to ensure individuals were working at or near their cognitive capacity as well as different types of (c) *disruption events* that interfered with goal activity. (Digital scenario design is described in more detail below.); (2) a semi-structured approach for training skill application and integration into goal pathways, including making use of digital cues to prompt SRS use at different cognitive junctures and providing data-driven feedback to guide and adjust skill use; and (3) methods for promoting skill transfer and generalization, including developing *experiential analogies* to identify opportunities to apply skills in daily life and establishing plans for prospective SRS use in these situations.

**Figure 2:**
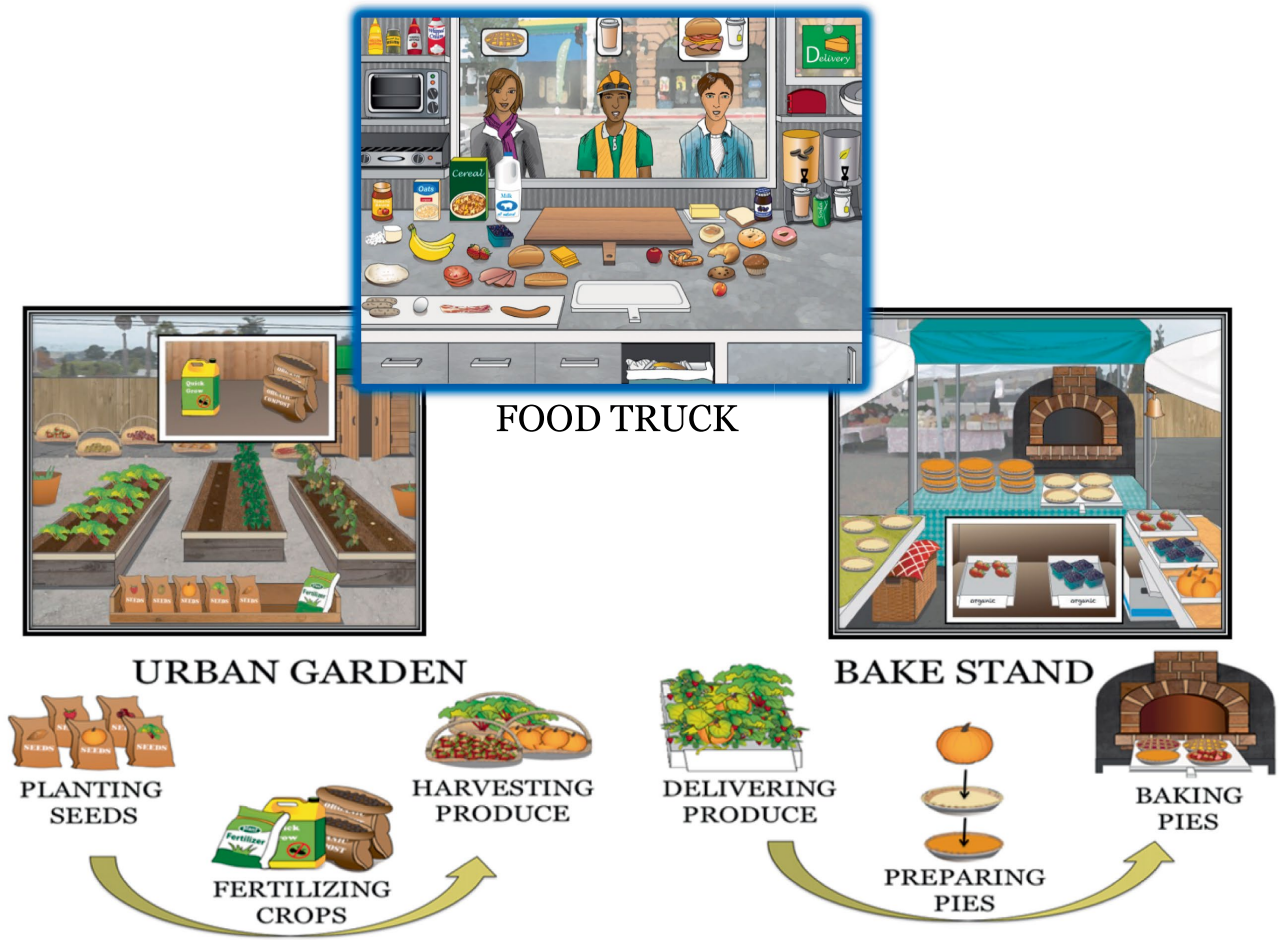
A representation of the three inter-connected digital scenarios. The top panel shows the primary scenario (Food Truck), which involves filling orders from memory. The bottom right panel depicts the second scenario (Bake Stand), in which the trainee prepares pies to supply the food truck. The bottom left panel depicts the third scenario (Garden), in which the trainee grows produce to be used in preparing pies. Multi-step action sequences involved with baking pies and growing produce are displayed underneath their respective panels. The coordinated management of these contexts requires the trainee to organize and execute multiple steps across scenarios, forming extended goal pathways comprised of varied neurocognitive processes.

**Scenario-based experiences and goal pathways:** We developed three distinct but inter-linked digital scenarios (see Figure 2) that incorporated different neurocognitive challenges to form a variety of unique goal pathways. Participants practiced applying SRS at distinct junctures along these pathways and integrating skill use into these broader goal contexts, a process that involved tailoring skill use to best serve the current demands and situation. We designed these scenarios to include several features to help maximize skill learning and generalization, including adopting a first-person perspective for all interactions (such that the trainee was an active agent in the scenarios, better connecting to personal life); incorporating tutorials to guide skill application in relation to different challenges and demands; setting specific skill use goals as well as reinforcing behaviors via immediate and summary feedback; actively cueing skill use during initial phases of learning, followed by fading of cues to encourage self-initiation of skills; adaptively adjusting select goal challenges based upon performance; and progressively increasing the complexity of goal pathways over training. Importantly, all goal-based challenges involved action-oriented cognitive processing and responding (e.g., responses requiring constituent elements to be combined, as in assembling a sandwich, in contrast to responding via a simple button press) and often required the integration of multiple neurocognitive abilities. These features are illustrated with an exemplar below.

Digital scenarios followed the storyline arc of opening and expanding a food truck business. The primary challenge extending across all three digital scenarios involves selectively encoding and processing customers’ orders that appear briefly as visual icons before fading (orders are placed and fulfilled in Scenario 1: Food Truck) and then serving them from memory. Individual items require one to four action steps to assemble; each customer orders between one to four items; and each order (trial) sequence contains three customers’ orders (resulting in a total memory set of four to 12 items, with each item composed of varying numbers of individual steps). Overall *information load* is individually adjusted (upward or downward) based on performance and progressively increases in complexity over training to include a greater proportion of multi-step items in customers’ orders as well as items that involve delays and timely follow-up (e.g., removing a pizza from the oven before it burns).

As training progresses, two more scenarios are introduced that extend the existing goal pathway, requiring participants *coordinate* and *follow-through* on additional cognitive actions while continuing to manage their workload in the Food Truck. First, players prepare and bake pies at a Farmer’s Market (scenario 2) to be served in the Food Truck; and second, players grow produce in an Urban Garden (scenario 3) that is used to make pies. These additional challenges require participants coordinate and follow-through on a series of steps (i.e., assembling and baking pies; planting and harvesting produce) within and across scenarios in order to maintain an adequate inventory of pies to meet customers’ demand. Trainees are present in only one scenario at a time (represented on the full-screen) and must remember to switch between scenarios to follow-through on steps.

An additional cognitive challenge that involves taking unique actions (e.g., using special vs. default ingredients when baking pies for select customers) in accordance with a higher-order goal (building a customer base for your future restaurant) is superimposed upon this basic goal pathway structure; opportunities to take goal-based actions are available within each scenario. Thus, this challenge requires participants actively retrieve and reconnect to a higher-order goal to guide individual responses at multiple points along an extended, multi-step goal pathway, all while continuing to manage increasing workload demands as described above.

Finally, one of the most important features of our experiential design was to *disrupt* goal-based cognitive processing at various junctures. Disruptions (Ds) were intercalated into goal pathways in the form of passersby who appear unexpectedly and make side requests, forcing participants to stop their current goal-directed activity, *re-direct* their attention to address the disruption, and then re-direct their attention back to the primary goal pathway. Ds occurred pseudo-randomly in each of these different scenarios, with variable and progressively increasing cognitive processing and time demands.

In sum, multiple inter-linked scenarios were designed that form unique goal pathways involving the integration of multiple neuro-cognitive functions, including encoding and retaining information; protecting information in working memory; following-through on multi-step tasks; sequencing and/or switching attention to follow-through on actions across scenarios; making choices based off of higher-order goals; and re-directing neural resources as needed to manage disruptions and complete goal pathways. SRS was learned through systematic and intensive application and integration along these pathways.

**Figure 3:**
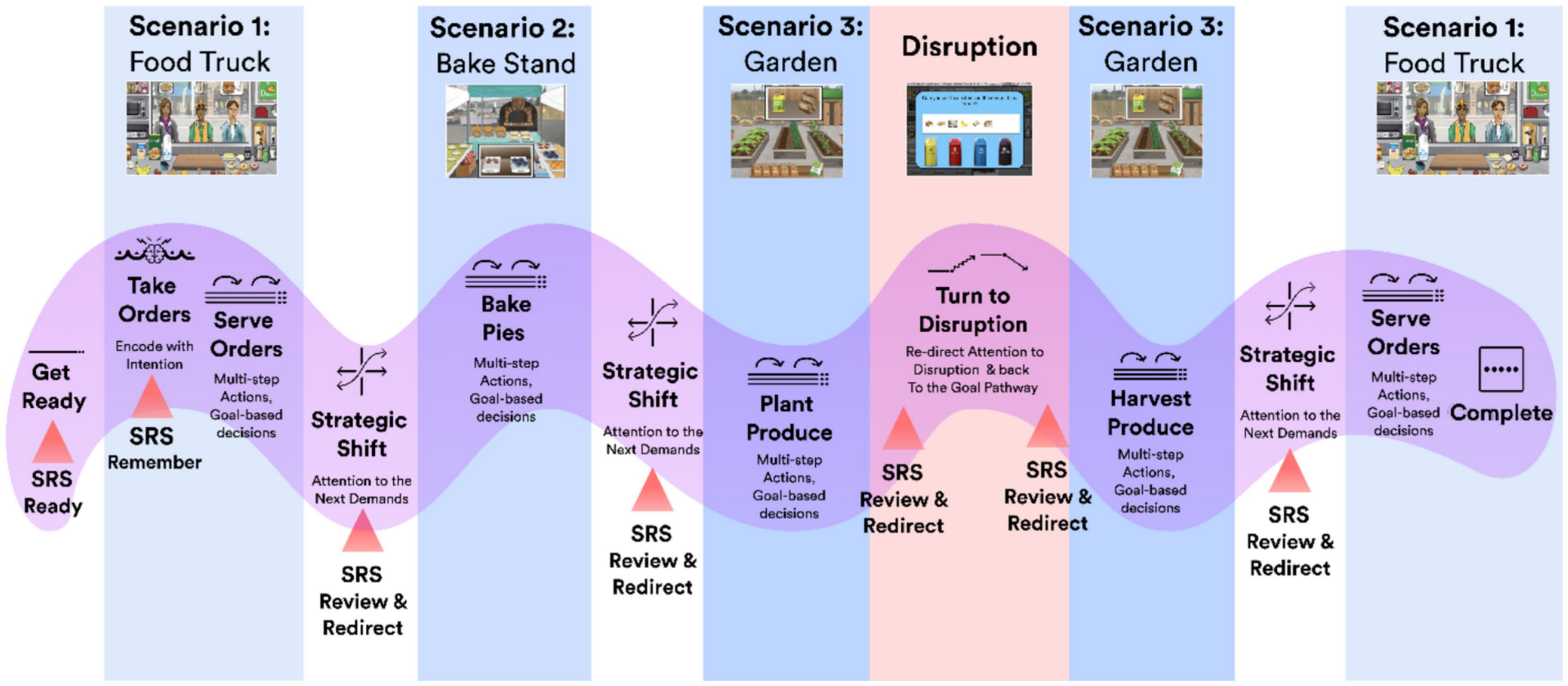
Remotely deployable digital scenarios are designed to provide opportunities for application and integration of SRS to goal-directed neuro-cognitive processes along a range of goal pathway experiences. The design of a complex goal pathway for a single trial is presented as an exemplar (see main text for details). Work demands translate to cognitive processing demands embedded along the goal pathway (represented in text along a pathway in the middle layer of the figure, progressing from left to right), occurring in the setting of each of the digital scenarios (with scenarios arrayed in the top layer of the diagram). Key opportunities for SRS application and integration to the cognitive processes are called out (bottom layer).

Figure 3 presents an example of a complex goal pathway depicting the coordination and sequencing of multiple cognitive activities across all three scenarios, including managing a disruption, and highlights opportunities to practice skill application and integration.

Prior to taking customers’ orders, participants practice regulating state (i.e., entering into a state of relaxed readiness) in preparation for the upcoming cognitive activity (SRS-Ready). Trainees are also coached to use the preparation phase to prospectively establish plans for using SRS to facilitate cognitive actions during the work they anticipate performing. Next, participants practice regulating state while taking orders; here, emphasis is placed on *encoding with intention* (SRS-Remember) and processing information to organize subsequent action plans (for fulfilling orders). Participants next start to fulfill customers’ orders, a process that requires maintaining and organizing information in working memory and responding accordingly. Simultaneously, while fulfilling orders, participants face two additional challenges: first, they must maintain the pie inventory, which requires coordinating and sequencing cognitive actions across the garden (planting and harvesting produce) and bake stand (preparing and baking pies) scenarios. Participants practice regulating state and reviewing actions plans for work to be completed before and after redirecting attention between scenarios/tasks (SR-Review and Redirect). Second, players are challenged to remember to provide special services to select customers based upon a higher-order goal; this process also requires that they reconnect with goal intentions and adjust their behaviors accordingly. Finally, while navigating this goal pathway, trainees encounter a passerby (disruption event) who makes an irrelevant request; this provides trainees another opportunity to practice regulating state and reviewing action plans prior to re-directing attention to the disruption, as well as after having resolved the disruption to effectively “get back on track” (SR-Review and Redirect).

Training followed a standardized sequence of experiences that centered on SRS application and integration to specific cognitive activities, but that allowed for individualization of content based upon each trainee’s personal motivations and relevant life situations, goals, and challenges. Scenario content and digital challenges were introduced in a progressive manner through a series of lessons, specifically to support learning objectives along the following sequence. Each learning objective includes an SRS application intent, along with a corresponding digital scenario challenge context that features a particular cognitive processing demand (as the opportunities and experiences for skill use), as well as personalized real life analog(s)(below, exemplars are provided, while the actual analogs would be individualized). By the end of training, each individual progressed through the entire lesson sequence, building to the most complex digital challenge scenario incorporating opportunities for all of the possible SR applications. This training sequence provides the experiential foundation to draw lessons from, establishing strong connections to the complex, dynamic contexts of personal life.

*Learning objective: SR-ready* - to achieve an optimal state prior to commencing goal-directed cognitive activity. *Digital scenario application*: Prior to starting the trial (prior to taking customers’ orders). *Real life analog example*: Prior to starting homework.

*Learning objective: SR-remember* - to facilitate intentional encoding of information relevant to upcoming cognitive work. *Digital scenario application:* While actively encoding customers’ orders. *Real life analog example:* When listening to instructions relevant to completion of homework.

*Learning objective*: *SR-ready and review* - to review situations with multiple or nested goals within a given context in order to guide decision making and appropriate action sequences. This involves making use of implementation intentions (see below) to perform certain actions, including cuing SRS at specific juncture or opportunities. *Digital scenario application:* Prior to starting a trial and while using SRS-Ready to prepare for cognitive activity, establish goal intentions for providing special services to select customers (a secondary goal)*. Real-life analog example:* Prior to starting homework and performing SRS-Ready, establish goal intentions for making flash cards for difficult-to-comprehend topics (secondary goal).

*Learning objective: SR-review and redirect* - to review action plans and goal contents when switching from one step to the next, and especially following a disruption. *Digital Scenario application:* Prior to switching to a disruption; after completing a disruption but before switching back to the main task; when switching between different challenge scenarios*. Real-life analog example:* Prior to switching attention from homework to take a planned break; when resuming activity after a planned break.

**Training skill application and integration:** Training was designed to support the development of a basic skill in goal-directed SR by providing myriad opportunities to apply and integrate SRS into different types of challenge contexts, specifically targeting a wide range of cognitive processes. Two general categories of SRS application were emphasized: first, *prospective* application of SRS to facilitate goal-directed functioning based on an understanding of upcoming cognitive processing challenges, and second, habitual *responsive* application of SRS to manage moments of dysregulation (e.g., in response to feeling frustrated or overwhelmed by goal-based challenges).

Trainers coached trainees on the application and integration of SRS into goal pathways. As noted, didactics and discussions helped to identify specific opportunities along goal pathways to apply SRS, as well as guide appropriate “next steps” following skill application. This process involved highlighting how various neuro-cognitive processes (e.g., attention, working memory, re-direction) contribute to individual goal pathways, and how the sequencing and coordination of these processes can be negatively impacted by disruptions. Having identified these points of vulnerability, participants were guided to form *implementation intentions* (Gollwitzer, 1999) to help self-cue skill application in these circumstances. This process involved simple and explicit “if (when)/then plans” for applying SRS in response to specific types of goal-based challenges, internal (dysregulated) experiences, or anticipated obstacles. A common example of an implementation intention in relation to digital challenges: “*If* a passerby disrupts my work, *then* I will use SRS to regulate my state *and* identify my next steps along my goal pathway *before* I switch my attention to the disruption.”

A digital button interface was designed to assist with training skill application as an automatic (contingent) response to goal-based challenges, as well as to achieve quantitative tracking. SRS was initiated with a button press in every instance of practice. This action temporarily suspended game activity while the individual attempted the linked SR application and integration into the specific action context; and once ready to resume activity, participants hit the button again to restart activity.

Participants practiced a general SR approach, with small modifications based upon the specific cognitive action context in which the skill was applied or the specific needs of the individual. Broadly speaking, this process involved initiating a SR response via controlled breathing (typically slowing down one’s rate of breath) until achieving a “relaxed and ready” state optimal to the task or challenge at hand. Importantly, participants were not prescribed a rigid behavioral method for regulating state but instead were trained to use experiential criteria [i.e., physiologic-experiential indicators of arousal (energy), anxiety (emotion), and attention (cognition)] to determine when they were “relaxed and ready.” These physiological-experiential qualities were introduced as existing along a continuum, with their optimal levels representing the apex of an inverted u-shaped curve (Nieuwenhuis, 2024) – that is, participants aimed to optimize qualities in which either insufficient or excessive amounts are associated with less-than-ideal functioning. Finally, as a means of providing an introduction to the process of self-introspection needed to help regulate state, participants also practiced five-minutes of state regulated breathing (in the absence of goal-based challenges, not directed to any cognitive actions) prior to starting training activities.

Overall, the digital experiential training framework provides multiple design elements to support skill learning, application, and integration: numerous cognitive challenges embedded within goal pathways, supportive cues for prompting SRS attempts in strategic contexts, highlighting failure opportunities for discovery-based learning, guidance to reinforce learning, and data-driven feedback to adjust behavior. Participants thus were provided numerous opportunities to apply and integrate SRS into the goal pathways and challenges described.

**Cultivation of transfer and generalization:** As discussed in greater detail above, coaches fostered connections between scenario-based experiences to other contexts in personal life via generalizable lessons, and, specifically the generation of *analogies* to personal goals and everyday life situations. Building upon this framework, coaches also helped form *implementation intentions* for applying and integrating SRS into personal life. In addition to helping cue SRS, implementation intentions emphasized prospectively formulating how SRS would be applied to neurocognitive demands. For example, trainers may help trainees extend implementations for dealing with disruptions in digital scenarios to being disrupted while studying for an exam: “If I am disrupted when studying, I will SRS and review my present goals/activity before restarting.”

### Study protocol

**Figure 4:**
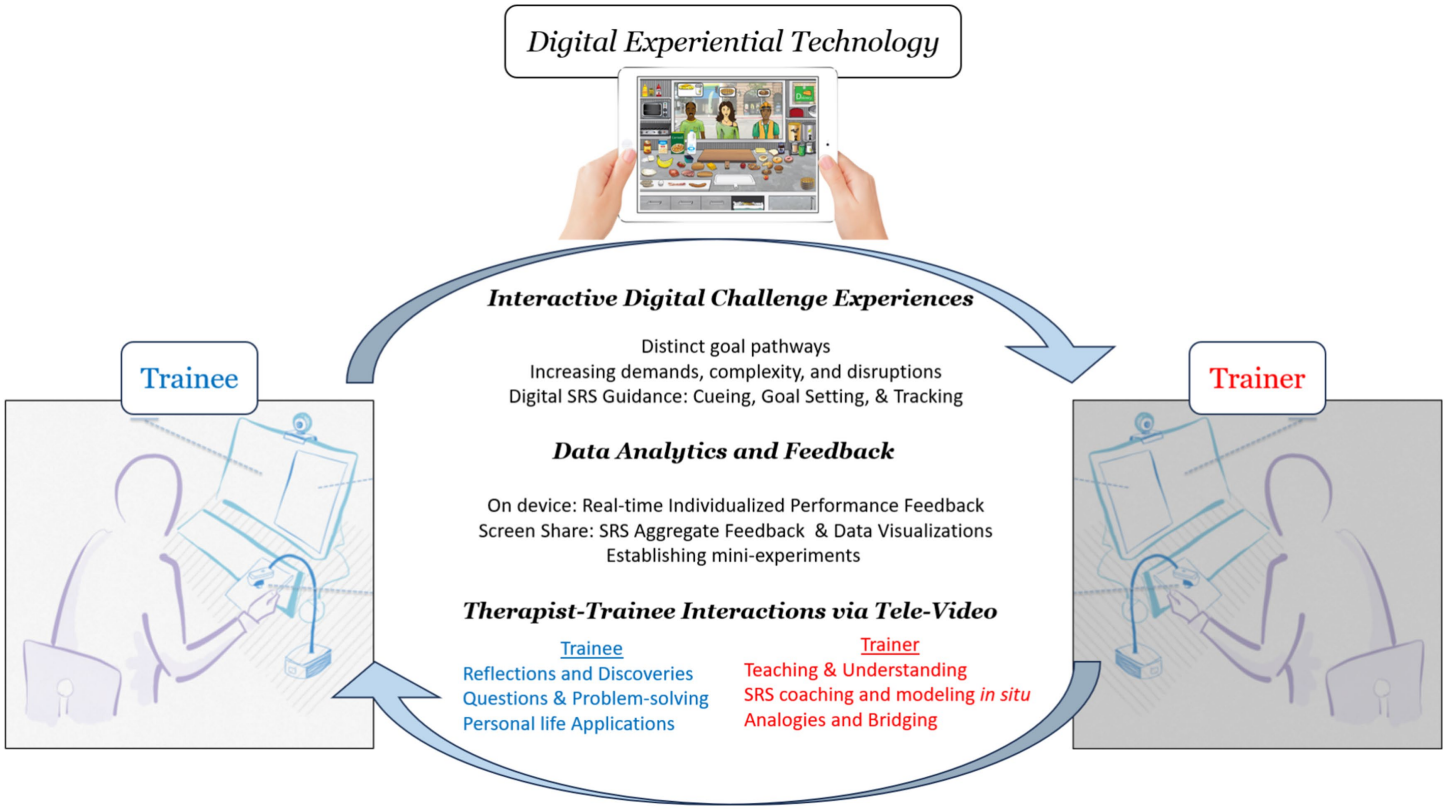
Schematic diagram highlighting multiple trainee-trainer-technology interactions in support of remote, guided experiential skill learning. (i) Interactive digital challenge experiences facilitate remote practice applying and integrating state regulation skills into complex goal pathways of varying kind, intensity, and complexity. Digital scenarios also include various supports to facilitate remote skill learning (e.g., tutorials, goal setting); (ii) Data analytics and feedback enable trainer-trainee dyads to examine the effects of skill use on various performance metrics, facilitating individualized guidance and coaching efforts; and (iii) Dyadic interactions via tele-video support multiple training objectives, including didactic instruction; problem-solving and optimizing skill use; and bridging training experiences to personal life.

The intervention consisted of seven, two-hour remote training sessions, weekly brief phone check-ins between tele-video meetings, and approximately 15–20 h of out-of-session skill practice. Participants were instructed to practice SR breathing for 5–10 min and skill application in digital scenario-based experiences for 20–30 min, five times per week. Training followed a manualized protocol and was completed within a 10-week span. All training was supervised by a PhD level neuropsychologist with expertise in TBI rehabilitation, with training itself conducted by this supervisor as well as professional trainees in psychology and social work with prior experience working with individuals with brain injury. Brief, scripted phone check-ins were conducted between training sessions to reinforce skill use, including linking game experiences to personal life goals and settings. Tele-video sessions were administered over two computer terminals in separate rooms within a VA campus or from VA to University of California. Each terminal was equipped with Cisco “Jabber” video-teleconferencing software, speakers, a web-camera, a separate stationary document camera, and an iPad with game software. The document camera was utilized to observe behavior in digital scenarios in real-time, while the web-camera relayed trainer-participant interactions. A technician was present on the participant side for technical assistance but was not involved in training (see Figure 4).

### Treatment-as-usual

All participants assigned to the treatment-as-usual condition were enrolled in VA health care and were instructed to continue their involvement with any brain injury-related services they were receiving, reflecting routine clinical care.

### Measurements

Participants completed multi-level assessments to quantify goal-directed functioning, spanning neurocognitive, functional, and self-reported functioning in personal life. All assessments were completed at baseline and after the intervention period (8–10 weeks later).

Neurocognitive performance was scored based on age, and when available, education, ethnicity, and repeated administration norms. To reduce the number of comparisons and variability among tests, scores were standardized and averaged into composite scores to index particular domains of functioning. We have utilized this approach in our previous research (Novakovic-Agopian et al., 2021; Novakovic-Agopian et al., 2018; Arnemann et al., 2015). Functional and self-report measures were scored according to procedures detailed in their respective manuals.

#### Neurocognitive processes

We created a composite measure of complex attention and executive functions, consisting of the following measures: (1) Wechsler Adult Intelligence Test – 3rd Edition (Wechsler, 1997) Letter Number Sequence subtest, which required re-ordering mixed number and letter sequences of increasing length; (2) Auditory Consonant Trigrams (Stuss et al., 1988), which required recalling three consonants after counting backward by 3 s from a target number for varying lengths of time; (3) the total errors from the Digit Vigilance Test (DVT) (Kelland and Lewis, 1996), which required crossing out a target number embedded among other numbers; (4) the time and total errors of trials 3–4 of the Delis-Kaplan Executive Function Systems (DKEFS) (Delis et al., 2001): Color-Word (CW) Interference test. Trial 3 required participants name the dissonant color of ink a color word was printed in, and trial 4 involved switching between naming the dissonant color of the ink a color word was printed in and reading the color word; and (5) Trails B (Bowie and Harvey, 2006), which was a task of alpha-numeric set-shifting.

#### Functional performance

We utilized an ecologically-informative functional measure, the Goal Processing Scale (GPS) (Novakovic-Agopian et al., 2014), to measure goal-based functioning. This observed, timed procedure required participants to pursue a goal of making an informed decision via a multi-step process in a complex setting. Participants worked on this goal by gathering information on three different self-selected activities (e.g., destinations for a weekend getaway), compare their choices across multiple criteria, and then making a selection of a preferred activity. Throughout the task, participants are required to follow specific rules and manage unanticipated challenges (e.g., distractions). Participants gathered information by means of a computer with an Internet connection, as well as a telephone and local phonebook. Blind evaluators rated participant performance across multiple domains following manualized procedures. We utilized the total score (i.e., observed performance averaged across eight subdomains of functioning) in our analyses.

#### Self-reported functioning

In order to index goal-directed control functioning in personal life, we utilized the following subdomains of the Behavior Rating Inventory of Executive Functions (BRIEF) – Adult Version (Roth et al., 2014). Working Memory, Shifting, Planning and Organization, and Task Monitoring. Participants rated a series of questions within each domain with respect to how frequently (i.e., never, seldom, often) they experienced problems over the past month. Age-adjusted T-scores were utilized in analyses.

Complementing these ratings of current functioning, participants reported their subjective perception of *change* across multiple cognitive domains utilizing the Goal Processing Questionnaire (Novakovic-Agopian et al., 2011) (GPQ; 1 = abilities have worsened, 5 = abilities have stayed the same, 10 = abilities have improved). This included assessment of functioning related to planning, initiating behaviors, self-monitoring, attending to and keeping information in working memory, sequencing tasks, problem solving, executing tasks, and learning from past experiences.

We assessed constituent experiences reflective of design principles and process-level aspects of intervention implementation by reviewing trainer notes detailing their clinical experiences working with individual trainees, as well as trainee responses to rating scales and open-ended questions designed to help guide training and gauge training experiences. The trainee queries included questions reflective of therapeutic progress along the stages of skill learning. We monitored aspects of trainer and trainee experiences on a session-by-session basis; this included tracking trainee comprehension of specific training content, evaluating the range and quantity of skill application during digital practice, notating the generation of analogies as connections to personal life, querying skill application in personal life, identifying obstacles to skill use as well as factors influencing successful skill application, and, finally, perceived benefit of skill use.

#### Analytic plan

We examined data from remote training to gauge constituent experiences as indicative of design intentions. This involved reviewing trainer notes containing observations during the training protocol as well as trainee in-session feedback forms. When reviewing these data, we reviewed materials with the goal of identifying and extracting experiences informative of the skill learning process and design intentions. We extracted and present experiences in terms of direct exemplars and anecdotes that are indicative of barriers, challenges, and successes for core aspects of intervention design, with a particular focus on characterizing interactions with the experiential technology design. The intention of this process was to evaluate constituent experiences of the implementation of training and design principles for achieving experiential learning and transfer and generalization of skills. This qualitative analysis process focused especially on to what extent and how prescribed digital experiences helped to bridge from training to real-life goals and life situations. We also quantified skill application during gameplay by examining SRS button usage.

We next examined longitudinal change with respect to neurocognitive functioning, functional task performance, and self-reported functioning separately for intervention and treatment-as-usual groups. We first plotted pre-post intervention changes for each individual by intervention group to identify visual trends, and then proceeded to calculate standardized effect sizes for aggregate change scores by group to characterize the potential strength of intervention effects. Calculation and interpretation of effect sizes for dependent samples were based on procedures outlined in Cohen (Cohen, 1988). Given this was a pilot investigation with a small sample size, we did not perform hypothesis testing (Kistin and Silverstein, 2015).

## Results

### Observations of remote guided experiential SRS training

We provide illustrative vignettes, qualitative observations, and quantitative data to highlight aspects of remote intervention implementation in relation to specific design and training objectives.

#### Deepening understanding of training themes

As expected for any treatment intervention, we observed variability in participants’ understanding of training themes and concepts; ways in which participants processed, retained, and interacted with this information over the course of the intervention; and, critically, their ability to apply this information within the contexts of digital scenarios and personal life. Broadly, this included participants’ abilities to conceptualize and/or identify aspects of their internal experiences as reflecting dysregulated cognitive-emotional states; to understand the rationale underlying SRS use and its intended purpose; and to identify opportunities (across both digital scenarios and personal life) where SRS application may be useful. Participants appeared to benefit from having these training concepts reinforced through different modalities throughout training (from their initial presentation in didactic format to their illustration and subsequent practice within digital scenarios to their ultimate application in personal life).

One particularly salient observation was that digital scenarios appeared helpful with illustrating or demonstrating the relevance of training concepts (e.g., state regulation, goal pathways) to goal-directed functioning. That is, having multiple and varied opportunities to experience, first-hand, becoming dysregulated in direct response to different cognitive challenges, as well as actively regulating state to facilitate cognitive function while challenged, appeared critical with transforming these abstract, propositional training concepts into embodied, action-oriented knowledge. Furthermore, intensively and systematically working through myriad *dysregulation-regulation* experiences appeared to help participants recognize and conceptualize their own subjective experiences as reflecting a dysregulated-regulated state, as well raise their awareness for the types of challenges they were individually susceptible to becoming dysregulated.

For example, one participant had difficulty comprehending the SR concept at the start of training, especially its potential relevance to his own individual experiences within digital scenarios and in personal life. His responses to initial in-session questionnaires gauging his basic comprehension of this concept was underdeveloped and incomplete, and he also had difficulty applying this concept to hypothetical situations (e.g., identifying instances in hypothetical interactions that may result in a dysregulated state). Experiences with digital scenarios helped improve this participant’s understanding of the SR concept as well as how SRS may improve his functioning; his trainer helped him examine how his own reactions to certain digital interactions (e.g., becoming emotionally activated in response to negative customer feedback) reflected a dysregulated state in which skill use may be helpful to remedy; establish plans for applying SRS at these times; and reflect upon his experiences (and changes to performance) with using SRS in these instances. This experience-informed conceptualization of SR, in turn, helped him to better identify analogous experiences with being dysregulated in personal life. Once he achieved this “insight,” he was better able to make use of the dysregulation concept in hypothetical situations, digital scenarios, and personal life.

Digital scenarios were also successfully utilized to strengthen understanding of the goal pathway concept, including that goal pathways are comprised of various neurocognitive processes that require sequencing and coordinating over time. Concepts such as working memory, encoding, and attentional re-direction (e.g., following disruptions, while coordinating multi-step follow-through tasks) that were first introduced in didactic format were observed to take on more personally-relevant meaning following interactions with digital scenarios. This further appeared helpful with translating these concepts into points of intervention; that is, engaging directly with these concepts through interactive scenarios appeared to help participants better identify these processes in the context of their individual goal pathways. For example, one participant observed how his cognitive abilities (e.g., regulating attention) were negatively impacted by the anxiety he experienced in anticipation of, as well as in response to, being disrupted; and how SRS helped to lessen this anxiety so he could more effectively re-direct his attention in a goal-directed fashion. With this experiential knowledge, he then started to conceptualize his day-to-day anxiety as a type of internal “disruption” that interfered with various aspects of functioning, including his work performance. He utilized this understanding to inform his use of SRS to redirect his attention when anxious thoughts started to distract him at home and at work. (See below for other examples of similar bridging experiences).

#### Use of scenarios to facilitate experiential skill learning

An overarching goal for the design and use of digital scenarios in training was to facilitate experiential skill learning. In particular, our intentions were to increase the quantity of opportunities to practice skill application across a range of challenge contexts; track and quantify skill application attempts; establish empiric relations between skill use attempts and behavioral performance; and utilize this information as feedback to inform the skill learning process.

As an initial step to achieving these objectives, we successfully quantified skill practice during digital scenarios: all participants interacted with digital challenges for at least 45 min each week, with the majority of participants (6 of 8) having practiced for more than 2 h per week. During these practice sessions, all participants utilized SRS at least 40 separate times per weekly training module, with the majority (6 of 8) practicing SRS at least 100 times per training module. See Table 2 for additional details.

**Table 2:** Quantification of skill practice in digital scenarios.

Participant	Total SRS applications	Total SRS practice time (in mins)
1	1,094	869
2	892	748
3	200	856
4	2,371	1,318
5	3,102	1,474
6	206	682
7	526	427
8	551	231
Median	722	802

Time was rounded to nearest whole number. SRS, State regulation skills.

Importantly, we observed that experiences with digital scenarios, supported by data-driven feedback and active guidance arrayed in progressive loops of learning, were generally helpful and effective with deepening understanding for how SR may facilitate goal-directed functioning. At the outset, participants typically started training not attempting to apply skills, often missing opportunities when and where skill application could be helpful. For example, analogous to personal life, during the experience of fast-paced demands in the digital scenarios, participants often did not recognize specific cognitive junctures and/or processes that could benefit from improved SR. Therefore, therapist-led coaching efforts were in large part dedicated to helping participants identify opportunities to practice SRS as well as formulate implementation intentions and set skill practice goals. The combination of digital features (e.g., cueing skill use at specific junctures) with explicit coaching appeared helpful with achieving robust skill practice.

However, the biggest positive contribution to training skill use appeared to be empiric feedback on the impact of SRS on performance within digital scenarios – experiences that, in turn, helped guide skill application in personal life. Trainer-trainee dyads successfully utilized the experimental framework to test specific hypotheses about the impact of skill use on performance, and this process appeared helpful with identifying instances in which skill use was beneficial on an individual basis. For example, one participant learned that he benefited most from using the SRS to switch attention between tasks in digital scenarios: he practiced regulating state while noting his place in the original task sequence (where he was in the task sequence and what worked remained) before switching his attention to the secondary task; and then repeating this process with each additional attentional shift. Another participant learned that he benefited most from using SRS in preparation for starting tasks (e.g., prior to each new trial in digital scenarios), particularly as a means of “resetting” and “clearing his mind” of the cognitive work he just performed so that he could better focus on new, upcoming work. He further learned that he tended to benefit from SRS use independent of his subjective levels of distress, or how he performed on the task immediately preceding it. This observation helped motivate him to work on establishing the routine of regulating state prior to starting new work, regardless if he was feeling dysregulated or not. Trainers worked with trainees on extending and integrating these scenario-based learnings into individual goal pathways (elaborated in more detail below).

A related way in which this empiric framework appeared to facilitate training was by enabling participants to address their skepticism about the SR approach in general as well as explore alternative approaches on an individual basis. For example, one unanticipated but nearly universal way this experimental approach appeared helpful was in challenging participants’ baseline skepticism about using SRS at all. When this approach was first introduced as the target skill, as well as when discussed specifically in relation to navigating digital challenges, many participants voiced the *lay theory* that using SRS would be counter-productive. That is, they expressed concern that any approach that involved “slowing down” to regulate brain state would negatively impact their ability to remember information and perform the task successfully. Relatedly, several participants reported that their initial attempts at utilizing SRS during digital scenarios felt unnatural and awkward, and, consequently they viewed the approach as unhelpful. In response to these beliefs and initial experiences with SRS use, several participants suggested that an opposite approach – one that emphasized “rushing” through the task as quickly as possible – would yield superior performances. We successfully made use of our data-driven framework to directly test these competing hypotheses. For example, trainers had participants directly test the effects of SRS vs. “rushing” strategies on their performance, routinely finding, contrary to participants’ predictions, that their performances generally improved when using SRS (in relation to both no skill use and rushing). These initial skill trial experiences, followed by data-driven feedback, contributed to trainees learning to put “intention” into a state of readiness for each cognitive action. Several participants acknowledged that they likely would not have tried SRS in personal life without first having experienced the positive benefits of its use in digital scenarios.

Finally, we observed the importance of having a wide range of challenge experiences of varying intensity for training SRS. For example, one participant’s skepticism about skill use was initially confirmed early during training, when the overall challenges were relatively simple; however, as training progressed and the challenges included managing multiple goal pathways simultaneously, the same participant observed strong performance gains associated with SRS use. That is, we observed SRS helped facilitate specific neurocognitive processes (e.g., encoding) in the context of increased goal complexity (in contrast to simply increasing the cognitive load) that were absent when goal pathway was less complex. This experience illustrated the relevance of the SR concept in relation to goal complexity as distinct from embedded neurocognitive tasks, per se.

#### Bridging from digital scenarios to real life: learning via analogies

A core emphasis of remote coaching was helping to explicitly bridge, or extend, participants’ learnings from their experiences with SRS in digital scenarios to goals and challenges in their personal life. This bridging framework appeared particularly helpful with identifying specific cognitive junctures within participant-identified goal pathways where improved regulation might be helpful. Participants reported the following examples: based on successful experiences with using SRS to cultivate a state of readiness for cognitive work, one participant extended this practice to using SRS in the car prior to the start of each day at work, as well as when starting new work assignments; another extended experiences with using SRS to process and encode information in digital scenarios to using SRS while attending to academic lectures and studying course material; and another participant extended experiences with using SRS while multi-tasking in digital scenarios to shifting between multiple domestic tasks and childcare responsibilities.

Additionally, trainer-trainee dyads made use of the negative emotional experiences during digital scenarios to address similar types of emotional challenges in personal life. For example, one participant who benefited from using SRS to temper strong reactions to negative customer feedback established plans to utilize SRS to better manage anticipatory anxiety associated with interacting with his boss at work, as well as to help mitigate negative emotional responses to challenging interactions with colleagues. Importantly, for this individual, the discovery that his dysregulation had to do with interpersonal sensitivities to receiving negative feedback was directly related to his experiences in digital scenarios, and this insight enabled him to identify parallels in personal life that otherwise may have been missed.

By the end of training, participants reported utilizing and benefiting from SRS in various situations in daily life where their cognitive-emotional state was challenged. Examples included use of SRS to navigate the various steps associated with repairing a vandalized car; when dealing with strong emotions and challenging interactions with a partner as they worked through a separation; to refocus attention and be less overwhelmed while teaching and interacting with students; to manage anxiety and remember names at a professional conference; and to improve focus during academic work. The repeated practice in the digital scenarios was reported to have helped participants more effectively use SRS in new situations.

### Intervention effects on multiple levels of goal-directed functioning

Summary of longitudinal results spanning multiple levels of goal-directed functioning are summarized in Table 3.

**Table 3:** Summary of pre- to post-training changes on transfer outcomes.

Variable	Baseline	Post-intervention	*d*
	M (SD)	M (SD)	
*Neurocognitive*			
Attention/executive functions composite			
SRS Training	0.14 (0.63)	0.54 (0.63)	0.64
Treatment-As-Usual	−0.03 (0.41)	−0.06 (0.57)	−0.07
*Functional performance (GPS)*			
Total score			
SRS training	8.00 (0.47)	8.25 (0.98)	0.41
Treatment-as-usual	7.81 (1.30)	7.79 (1.45)	−0.02
*Ratings of functioning (BRIEF)*			
Working memory -*T*			
SRS training	73.00 (15.13)	69.13 (15.08)	−0.55
Treatment-as-usual	72.75 (23.16)	68.25 (20.67)	−0.09
Set shifting – *T*			
SRS training	62.13 (10.37)	58.88 (8.18)	−0.44
Treatment-as-usual	66.38 (15.40)	60.75 (15.05)	−0.27
Planning and organization – *T*			
SRS training	71.88 (16.37)	67.75 (13.19)	−0.56
Treatment-as-usual	65.00 (21.59)	58.50 (21.59)	−0.04
Perception of changes to functioning (GPQ)*			
Attention and working memory			
SRS training	–	7.59 (0.90)	2.88
Treatment-as-usual	–	4.52 (1.14)	−0.42
Sequencing			
SRS training	–	6.92 (0.93)	2.06
Treatment-as-usual	–	4.76 (0.86)	−0.28
Planning			
SRS training	–	7.21 (0.85)	2.60
Treatment-as-usual	–	4.68 (1.27)	−0.25

SRS, State Regulation Skills; GPS, Goal Processing Scale; BRIEF, Behavior Rating Inventory of Executive Functions. Higher BRIEF scores reflects increased dysfunction. GPQ, Goal Processing Questionnaire. *Scale anchors: 0 = Function worsened since study entry; 5 = No change to function since study entry; 10 = Function improved since study entry. Cohen’s *d*: 0.02 = small, 0.05 = medium, 0.08 = large.

#### Neurocognitive tests of attention and executive functions

**Figure 5:**
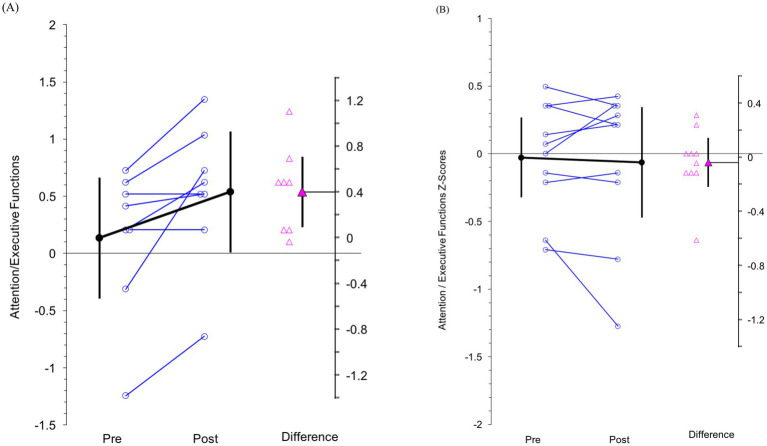
Individual pre- and post-training scores (open circles) and individual change scores (open triangles) for the primary composite outcome reflecting complex attention and executive functions for participants allocated to **(A)** remote training of state regulation skills and **(B)** treatment-as-usual conditions. Aggregated pre-post scores (filled circles connected via bold line) and change scores (filled triangle) are also shown.

Participants’ performances improved moderately on a composite measure of complex attention and executive functions following remote training (d = 0.64) but not treatment-as-usual (d = −0.07). Figure 5 shows these differential rates of change for each participant by training condition.

#### Ecologically-informative measure of goal-directed functioning

Participants’ performances on a complex, ecologically-informative task of goal-directed functioning (the Goal Processing Scale) also improved modestly following remote training (d = 0.41); participants’ performances in the treatment-as-usual condition, by contrast, remained unchanged over time (d = −0.02).

#### Personal life functioning

*Changes on the BRIEF*. Relative to reported functioning at baseline, participants in the remote training condition reported less everyday problems with attention and working memory (d = −0.55), shifting between tasks (d = −0.44), and planning and organization (d = −0.056). On the contrary, participants in the treatment-as-usual condition showed no reduction to problems with attention and working memory (d = −0.09) and planning and organization (d = −0.04), but they did report small improvements with set-shifting abilities (d = −0.27).

*Perceived changes on the GPQ*. At the conclusion of the study, participants rated how much they perceived specific cognitive abilities to have changed following the interventions. Participants in remote training reported large improvements to attention and working memory (d = 2.88), sequencing (d = 2.06), and planning (d = 2.6). By contrast, treatment-as-usual participants reported their abilities in these domains worsened slightly (attention and working memory: d = –0.42; sequencing: d = −0.28; and planning: d = −0.10).

## Discussion

With remote rehabilitation becoming an increasingly important part of clinical care for individuals with brain injury, there is a pressing need to develop intervention approaches that specifically address gaps in the skill learning and generalization process, especially when individuals and their providers are in separate locations. In this report, we relayed an important step in the iterative development and testing of a remotely deployable, technology-augmented approach to guided experiential learning of SRS. In this approach, individuals learn to apply and integrate skills into a range of goal challenges, starting with purpose-built remotely deployable digital challenge scenarios and bridging toward application in personal life, while supported by remote guidance to facilitate skill learning and generalization. Here, we reported on a pilot implementation, relayed “user experiences” with training (emphasizing contributions of training design to advance clinically-relevant skill learning objectives), and shared pilot data informative of the potential for this approach to improve goal-directed functioning for individuals with TBI.

We designed and implemented training to address a series of inter-related components important for skill learning and generalization in remote rehabilitation. On the one hand, this included facilitating intensive skill practice along a range of goal pathways with embedded neurocognitive demands (e.g., selective attention, working memory, re-direction of neural processing to achieve follow-through and manage disruptions); and on the other hand, this included supporting skill development and generalization to daily life via remote coaching. We integrated digital scenarios into remote sessions to function as “teachable encounters” to help deepen understanding of training concepts, guide effective application and integration of skills into cognitive processing in contexts of goal-based challenges, promote meta-cognitive awareness of individual vulnerabilities as opportunities to use SRS, and help identify generalizable lessons from training experiences to support skill application in personal life. The guiding rationale underlying this approach was that therapist-guided, technology-augmented experiential skill training, anchored in digital scenarios, would form a foundation upon which skill use could then be bridged to personal life goals and settings.

Remote rehabilitation implementation experiences indicated that the integration of all aspects of technology into training was feasible and highly valued by constituents. Both trainers and trainees reported benefiting from being provided with *concrete learning opportunities* (via digital scenarios) that were individually calibrated and reiterated across deepening loops of learning. In particular, constituents valued having a range of controlled opportunities, conveniently accessible on demand, to practice SRS in relation to different neurocognitive demands embedded along goal pathways. In addition, constituents were able to productively engage with digital data and feedback remotely. Providing participants a range of concrete opportunities to practice SRS while receiving digital and trainer-led feedback and guidance appeared to facilitate skill learning and generalization.

The intentional design and integration of technology into remote rehabilitation with clear, explicit contributions to the skill learning process represents an important clinically-relevant advance. Without the integration of remotely-deployable technology, participants would not have been able to practice SRS as intensively, systematically, or in as a wide a range of contexts, nor benefited from data analytic feedback to guide skill development. The experiential technology enabled participants to accumulate substantial practice with applying SRS in different contexts; achieving (and tracking) this quantity of skill practice is noteworthy and something that is difficult to achieve without technology, despite the importance of repetition for developing new skills as habitual or automatic responses when experiencing challenges (Kimberley et al., 2010). For individuals with brain injury, in particular, overlearning a behavior (e.g., using SRS when overwhelmed by cognitive processing demands) may be necessary to override deeply ingrained and often maladaptive modes of responding (e.g., shutting down).

Digital scenarios also supported participants in the learning process by providing environments encouraging of skill practice. The use of digital scenarios helped to address a range of fundamental issues that may otherwise impede or slow learning, such as difficulty finding suitable opportunities for skill practice, difficulties with integrating skills directly into inherently challenging life situations, reticence about trying a new skill given the potential risk associated with failures (especially during early phases of skill learning), and a lack of confidence about one’s ability to effectively utilize the skill.

In addition to these aspects of intentional design, we also discovered advantages of digital scenarios in helping to address negative baseline beliefs about a new approach as well to overcoming ingrained behaviors. For example, some participants were skeptical about the recommended skill and believed that the opposite approach, rushing through the task as quickly as possible, would yield superior performance. These beliefs functioned as potential obstacles to learning, but by using digital scenarios as training opportunities, participants were able to test these beliefs without concern of incurring personal costs (e.g., a failure in a personally-significant challenge setting). Participants were able to observe and reflect on the contrasting effects of different approaches, benefiting from data analytic feedback. This provided a solution to the challenge of early failure experiences discouraging ongoing skill development (e.g., “I tried the skill at my job, and it made things worse!)

Experiential learning technology supported a vital and novel therapeutic approach to training SRS: practice integrating regulation of brain state directly into the contexts of goal-based cognitive-behavioral action sequences. To our knowledge, this is the first demonstration of intervention design that incorporates remote experiential technology to train SR applied and integrated into “live” and dynamic cognitive processes embedded in complex goal pathways. This approach trained regulation of brain state as a neurocognitive skill, directly integrating it into attention, working memory and executive processing in naturalistic contexts of goal challenges. It is illustrative to contrast this with training approaches such as task practice alone (with no defined generalizable skill and using simplified, isolated tasks; e.g., Simons et al., 2016), SR practice in isolated settings (outside of functional contexts; e.g., Jha et al., 2007), as well as remote coaching alone (without scenario-based experiences, e.g., Ng et al., 2013). There is a gap for many individuals with TBI in transferring any partial learning to struggles embedded in daily life. Our pilot implementation demonstrated, however, that it is possible to train goal-directed, action-oriented applications of SRS in tele-rehabilitation to bridge steps toward generalization.

*Personalized data-driven feedback* contributed to learning of skill application and integration in valuable ways. At a very basic level, the technology-augmentation allowed tracking and recognition of SRS attempts. This directly supported learning efforts, including providing data to inform coaching guidance. Complementing this, data helped in identifying lack of skill use – this allowed trainers to raise awareness of *missed opportunities* and recast them as learning opportunities. We further observed that data feedback was critical in helping each individual optimize SRS use in different cognitive contexts, related both to specific embedded demands (e.g., while encoding information) as well as in relation to the overall complexity of the goal pathway. At a deeper level, *objective* data helped calibrate self-perception of the effectiveness of skill use, noting that self-awareness is often altered following brain injury (Dromer et al., 2021). Without objective tracking, inaccurate conclusions may lead to negative self-efficacy beliefs, reducing the likelihood that a new skill will be adopted in practice (Holladay and Quiñones, 2003). Data review was helpful with challenging biased observations and thereby promoting more accurate assessments.

A vital element of intervention design was integrating digital scenarios and data feedback into a system of remote coaching to support skill learning and generalization to daily life. In keeping with the intended design, training experiences formed important loops of learning: abstract concepts (e.g., goal pathways) were first introduced via psychoeducation prior to being illustrated and enriched via direct experiences in digital scenarios; after having achieved greater understanding of these concepts via concrete experiences, individuals were then better able to apply skills to novel situations and settings; and these experiences and learnings, in turn, contributed to subsequent skill learning and generalization as training progressed. For example, digital experiences deepened understanding of basic training concepts centered on specific cognitive processes (e.g., selective attention, working memory). Concepts were first introduced via standard didactic methods but were not always fully understood, retained across training sessions, or comprehended well enough that they could be flexibly applied to novel and personal situations. This process appeared valuable for concepts such as *goal pathways* and *cognitive junctures*, as well as myriad examples of how a dysregulated state may manifest in the context of goal pursuit. Illustrating these concepts experientially helped bridge to goals and situations in personal life – a vital step in transfer and generalization (Kolb et al., 2001).

In direct support of skill generalization, remote coaches were successful with utilizing digital scenarios as *analogies* to personal life. That is, therapists utilized scenario-based experiences to help identify parallels with goal-based challenges in daily life, identify personal vulnerabilities in the context of self-identified goals, reflect upon how to best utilize SRS in these situations to improve functioning, and establish a plan (i.e., implementation intentions) for using SRS at those times. Participants further engaged in discussions identifying contextual factors that contribute to dysregulation that might be improved by SRS, such as when feeling overwhelmed, frustrated, and discouraged when mistakes are made and result in negative or critical feedback. Overall, scenario-based experiences appeared to facilitate participant identification of concrete ways skill use could benefit goal-directed functioning in personal life.

Ultimately, this intervention formulation sought to train the *self-cued* activation of SR, with scenario-based experiences as a critical steppingstone to effective skill use in personal life. Digital scenarios provided mechanisms to cue skill application as an initial scaffold that faded out as training progressed. Training emphasized the internalization of skills by establishing implementation intentions, especially as supported by goal frameworks, and extensive practice to increase automaticity of skill use in the context of goal-based challenges. Ultimately, multiple aspects of our intervention design converged to support the self-cued activation of SRS to better integrate into life situations.

To gauge the plausibility that training SRS applied and integrated to goal-based challenges could improve goal-directed functioning outside the training environment, we examined longitudinal changes across multiple levels. In general, in order to best understand the potential effects and limitations of any intervention, it is important to evaluate possible differential effects at different levels of functioning. It is worth noting that there may be disconnects between these different levels of functioning that are important to understand as part of advancing rehabilitation neuroscience. For example, specific neurocognitive processes may operate differently when integrated with other processes, such as is necessary to pursue complex goals. This may be one reason why trainings focused on task practice in relative isolation have resulted in limited transfer and generalization to different tasks and settings (Simons et al., 2016; Melby-Lervåg and Hulme, 2013). First, we found training-associated improvements on a composite measure of complex attention and executive functions, providing a preliminary suggestion of transfer to non-trained cognitive tasks. It is noteworthy that improvements on neurocognitive measures are not always observed following interventions intended to improve goal-directed functioning, such as those limited to psychoeducation or training metacognitive strategies without emphasis on experiential skill training (Krasny-Pacini et al., 2014; Kennedy et al., 2008). Second, individuals receiving remote training reported that working memory functioning in personal life improved following the intervention. These self-ratings were supported by specific anecdotes, reflecting successful application and integration of SRS into personal life. Third, training participants showed improvements in observed functional performance in complex, ecologically-informative settings, of moderate effect size. Generally, these effects were not observed for participants enrolled in standard care. The current results suggest that it is plausible for thoughtfully designed technology-augmented tele-rehabilitation training to change goal-directed functioning at proximal and distal levels, so further study is warranted. Indexing goal-directed functioning at multiple levels in future studies will help to inform rehabilitation neuroscience for any tele-rehabilitation intervention. The multi-level approach also creates a framework for studying relationships between levels, including investigating neurophysiology that may underlie functional changes (Chen et al., 2020; Tenenbaum et al., 2009).

Study limitations suggest important areas for further work. First, this proof-of-principle project implemented the intervention remotely, but assessments were conducted in person. Future studies would benefit from innovations in remote assessment methodology, allowing engagement of a broader audience in tele-rehabilitation. Second, this hybrid report is based on a small sample and with a comparison condition that was clinically-relevant but not standardized. Pilot data suggest it will be worth pursuing a randomized-controlled design that makes use of a defined active comparison condition and a larger sample of participants. Larger samples will also allow exploration of other relevant questions, such as the potential moderating effects of various cognitive and/or emotional factors at baseline on subsequent skill learning and neurocognitive outcomes. Third, this study was limited to measuring short-term effects, and future studies would benefit from conducting long-term follow-up of skill use behaviors and other training outcomes. Fourth, quantification of skill use was successful but limited to the digital scenarios. Future research could benefit from innovative approaches for better measuring skill use in personal life and directly linking skill use to changes in functioning along individual goal pathways. Potential options could include innovations in analog or digital tools to quantify SRS application in support of progress toward personal goal attainment, in naturalistic settings. Fifth, it is fundamentally difficult for many individuals to develop awareness of internal state, and the guided experiential learning approach could be further developed to maximize skill learning objectives. The clinically-applicable and experimenter-controlled intervention framework we developed provides a foundation for innovations that incorporate physiological monitoring and feedback linking physiological state with subjective internal experiences, helping to cue SRS as well as providing feedback on the success of skill use.

Finally, the SRS-AIRE framework was tested as a “standalone” approach as a proof-of-principle study, but it will be important for future research to consider the potential placement of SRS training in the broader context of TBI rehabilitation. Ideally, SRS training would not function as a standalone intervention, but would be utilized as a *readiness* intervention and then rationally combined with other approaches to maximize learning goals. This has potential to strengthen learning associated with other rehabilitation approaches (Chen et al., 2020; Chen and Loya, 2014), such as with any form of education or skill or strategy training [e.g., integrated directly into project-based meta-cognitive strategy training (Novakovic-Agopian et al., 2018)]. SRS readiness training could also cultivate readiness for other pursuits founded on learning processes, such as in school or in adapting to new employment.

In common clinical practice, approaches that focus on SR, such as mindfulness and meditation, are primarily used for developing emotional coping, relaxation, and stress relief. It is infrequently the case that SR approaches have been trained specifically to try to improve cognitive functioning. However, in our experiencing working with individuals with TBI, who may also experience PTSD and other common comorbidities, developing SRS is one of the most difficult aspects of rehabilitation. Many individuals have difficulties with SR practices in isolation due to factors such as distractibility, difficulty in understanding the utility of the approach, or experiencing intrusive and distressing thoughts. With respect to rehabilitation methodology, training SRS as an applied skill, integrated into goal-oriented action contexts, may more directly bridge the missing links from optimizing brain state to maximizing effective goal-directed functioning. The preliminary results of this pilot also support the plausibility that such bridging could be of benefit even in remote rehabilitation.

Overall, this study represents a significant innovation for remote neurocognitive skills training after brain injury: the intentional design of remotely deployable digital technologies integrated with remote coaching can provide highly individualized and personally-meaningful training experiences, and may help to strengthen specific neurocognitive abilities and improve goal-directed functioning broadly. Of vital importance, anchoring the intervention in a digital framework enriched the therapeutic environment and empowered trainers and trainees alike in ways that would not otherwise be possible in remote settings. This report provides an example of how digital technologies can be rationally integrated into rehabilitation interventions to be of practical use by first considering underlying learning principles that technology is intended to augment, and then translating these principles into clinically-relevant approaches to training that support rehabilitation goals. Further work is warranted.

## Data Availability

The raw data supporting the conclusions of this article will be made available by the authors, without undue reservation.
